# Fecal Microbiota and Gut Microbe-Derived Extracellular Vesicles in Colorectal Cancer

**DOI:** 10.3389/fonc.2021.650026

**Published:** 2021-09-14

**Authors:** Jihye Park, Nam-Eun Kim, Hyuk Yoon, Cheol Min Shin, Nayoung Kim, Dong Ho Lee, Jae Yong Park, Chang Hwan Choi, Jae Gyu Kim, Yoon-Keun Kim, Tae-Seop Shin, Jinho Yang, Young Soo Park

**Affiliations:** ^1^Department of Internal Medicine, Yonsei University College of Medicine, Seoul, South Korea; ^2^Department of Public Health Sciences, Graduate School of Public Health, Seoul National University, Seoul, South Korea; ^3^Department of Internal Medicine, Seoul National University Bundang Hospital, Seongnam, South Korea; ^4^Department of Internal Medicine, Chung-Ang University College of Medicine, Seoul, South Korea; ^5^R&D Center, Institute of MD Healthcare Inc., Seoul, South Korea

**Keywords:** microbiome, metagenome, gut microbe-derived extracellular vesicles, colorectal cancer, cancer stage

## Abstract

The human microbiota comprises trillions of microbes, and the relationship between cancer and microbiota is very complex. The impact of fecal microbiota alterations on colorectal cancer (CRC) pathogenesis is emerging. This study analyzed changes in the microbial composition in CRC subjects with both fecal microbiota and gut microbe-derived extracellular vesicles (EVs). From August 2017 to August 2018, 70 CRC patients and 158 control subjects were enrolled in the study. Metagenomic profiling of fecal microbiota and gut microbe-derived EVs in stool was performed using 16S ribosomal DNA sequencing. Relative abundance, evenness, and diversity in both the gut microbiota and gut microbe-derived EVs were analyzed. Additionally, microbial composition changes according to the stage and location of CRC were analyzed. Microbial composition was significantly changed in CRC subjects compared to control subjects, with evenness and diversity significantly lower in the fecal microbiota of CRC subjects. Gut microbe-derived EVs of stool demonstrated significant differences in the microbial composition, evenness, and diversity in CRC subjects compared to the control subjects. Additionally, microbial composition, evenness, and diversity significantly changed in late CRC subjects compared to early CRC subjects with both fecal microbiota and gut microbe-derived EVs. *Alistipes-*derived EVs could be novel biomarkers for diagnosing CRC and predicting CRC stages. *Ruminococcus* 2-derived EVs significantly decreased in distal CRC subjects than in proximal CRC subjects. Gut microbe-derived EVs in CRC had a distinct microbial composition compared to the controls. Profiling of microbe-derived EVs may offer a novel biomarker for detecting and predicting CRC prognosis.

## Introduction

Colorectal cancer (CRC) has become a global health problem because of the increasing incidence of CRC in young adults (1, 2). Western dietary patterns and obesity have been strongly linked to CRC development (3, 4). Various studies have suggested that the pathogenesis of CRC is influenced not only by genetic factors but also by gut microbial composition altered due to ingested food or environmental factors. Gut microbiota induce oxidative stress and DNA damage in response to chronic inflammation, cell proliferation, and the production of metabolites such as butyrate (5). In animal studies, *Fusobacterium nucleatum* is associated with CRC pathogenesis by expressing a bacterial cell surface adhesion component, which can bind to host E-cadherin (6, 7). Enterotoxigenic *Bacteroides fragilis* is enriched in human CRC, resulting in cell morphology changes, E-cadherin cleavage stimulation, and colonic barrier function reduction (8).

Efforts to develop microbe-based cancer therapy have attracted more than 100 years from Coley’s toxin in patients with bone cancer (9). Immunotherapy, including anti-programmed death receptor-1 (PD-1) inhibitors and anti-cytotoxic T-lymphocyte associated protein 4 (CTLA-4), provides a therapeutic response by modulating the gut microbiota (10, 11). Several approaches, including probiotics such as VSL#3 and LGG, could alter the gut microbiota composition (12, 13). Probiotics may regulate the immune system and inhibit the progression of CRC. Dietary changes by eliminating animal fat and a high-fiber diet may ultimately be a considered cancer therapy in some studies (14, 15). Nevertheless, the human microbiota comprises trillions of microbes, and the relationship between cancer and microbiota is very complex. Due to the heterogeneity of the microbiota, there are many limitations to finding therapeutic agents targeting specific microbiota.

Gut microbes, including gram-negative bacteria and some gram-positive bacteria, can produce extracellular vesicles (EVs), also called nanovesicles, and are upregulated during cell activation and growth during cancer development (16). Excess EVs can be released into the circulation, including plasma, saliva, gastric juice, and intestinal luminal liquid. There are many approaches for studying EV-associated RNA, membrane lipids, and proteomic composition of EVs (17, 18). EV-based early diagnostic biomarkers in patients with gastrointestinal cancer are challenging areas (19). Kanwar et al. developed a microfluidic device for circulating exosome characterization in patients with pancreatic cancer patients (20). Choi et al. found some proteins in colorectal cancer-derived EVs by proteomic analysis (21, 22). However, to the best of our knowledge, there have been very few reports on the development of CRC biomarkers by 16S ribosomal DNA sequencing metagenomic profiling with EV samples isolated from stool samples (23).

This study hypothesized that microbe-derived EVs interact with the gut microbiota and are associated with CRC development. Microbial composition changes in CRC subjects were analyzed and compared with control subjects of microbiota with both stool samples and microbe-derived EV samples. Additionally, differences in microbial composition were analyzed according to the stage and location of CRC.

## Materials and Methods

### Patients and Sampling

Between August 2017 and August 2018, 158 stool samples were collected from control subjects, and 70 stool samples were collected from colorectal cancer patients who visited the CRC clinic at Seoul National University Bundang Hospital (Seongnam, Republic of Korea) and Chung-Ang University College of Medicine (Seoul, Republic of Korea) before undergoing any treatment. The exclusion criteria were as follows: (i) patients who had been diagnosed with gastric cancer, colorectal cancer, or other malignant diseases in the past and had undergone surgery and chemotherapy; (ii) patients who had been diagnosed with gastric dysplasia or gastric adenoma; (iii) pregnant women; (iv) patients who had been taking antibiotics or probiotics within last three months; and (v) patients who declined to participate in the study. For the enrolled patients, extensive medical data were collected every time they visited our clinic using electronic medical records. Fecal samples were self-sampled and stored at -20°C, transported to the laboratory, and frozen at -70°C. Control stool samples were selected from a previously collected cohort of healthy patients above 40 years of age who had abdominal symptoms but were not diagnosed with irritable bowel syndrome. There was no overlap of participants in the control group between previous studies. This study was approved by the Institutional Review Board of Seoul National University Bundang Hospital (IRB No: B-1708/412–301). Written informed consent for the use of medical records was obtained from all the participants. 23 CRC patients (only fecal microbiota samples) overlap with those in previous reports (24). 31 CRC patients (both fecal microbiota and gut microbe-derived EV samples) overlap with those in previous reports (23).

### Extracellular Vesicles (EV) Isolation and DNA Extraction

Human stool samples were filtered through a cell strainer after being diluted in 10 mL of PBS for 24 h. The samples were centrifuged at 10,000 × g for 10 min at 4°C to separate EVs from stool samples. After centrifugation, stool sample pellets contained bacterial cells, and the supernatant of stool samples contained EVs. Bacteria and foreign particles were thoroughly eliminated from the stool sample supernatant by sterilizing the supernatant through a 0.22-µm filter. To extract DNA from bacterial cells and bacterial EVs, bacteria and EVs were boiled for 40 min at 100°C. To eliminate the remaining floating particles and waste, the supernatant was collected after 30 min of centrifugation at 13,000 rpm at 4°C. DNA was extracted using a DNeasy PowerSoil Kit (QIAGEN, Germany) according to the instructions of the manufacturer. DNA extracted from bacterial cells and EVs in each sample was quantified using a QIAxpert system (QIAGEN, Germany).

### Bacterial Metagenomic Analysis Using Extracellular Vesicles (EV) DNA

Bacterial genomic DNA was amplified with 16S_V3_F (5′-TCGTCGGCAGCGTCAGATGTGTATAAGAGACAGCCTACGGGNGGCWGCAG-3′) and 16S_V4_R (5′-GTCTCGTGGGCTCGGAGATGTGTATAAGAGACAGGACTACHVGGGTATCTAATCC-3′) primers, specific for the V3-V4 hypervariable regions of the 16S rRNA gene. Libraries were prepared using PCR products according to the MiSeq System guide (Illumina, USA) and quantified using the QIAxpert (QIAGEN, Germany). Each amplicon was quantified, set equimolar ratio, pooled, and sequenced on a MiSeq platform (Illumina, USA) according to the recommendations of the manufacturer.

### Analysis of Bacterial Composition in the Microbiota

Paired-end reads that matched the adapter sequences were trimmed using Cutadapt version 1.1.6, with a minimum overlap of 11, a maximum error rate of 15%, and a minimum length of 10 (25). The resulting FASTQ files containing paired-end reads were merged with CASPER version 0.8.2, with a mismatch ratio of 0.27, and then quality-filtered using the Phred (Q) score-based criteria described by Bokulich (26, 27). Any reads shorter than 350 bp or longer than 550 bp after merging were discarded. A reference-based chimera detection step was conducted with VSEARCH version 2.3.0 against the SILVA gold database (28, 29) to identify the chimeric sequences. Sequence reads were clustered into operational taxonomic units (OTUs) using VSEARCH with an open clustering algorithm under a 97% sequence similarity threshold. The representative sequences of the OTUs were finally classified using the SILVA 132 database with UCLUST (*parallel_assign_taxonomy_uclust.py* script in QIIME version 1.9.1) under default parameters (30). The original contributions presented in the study are publicly available in NCBI. Raw reads of the fecal microbiota and microbe-derived EVs for colorectal cancer patients were deposited into the NCBI SRA database, respectively (Accession Numbers: SRR15182562–SRR15182631; SRR15182632–SRR15182701). Raw reads of the fecal microbiota and microbe-derived EVs for control patients from three datasets were deposited into the NCBI SRA database respectively (dataset 1: SRR15056567–SRR15056766; SRR15056787–SRR15056992; dataset 2: SRR15244175-SRR15244358; SRR15245161-SRR15245345; dataset 3: SRR15204197-SRR15221118; SRR15243500-SRR15243683) (**Supplementary Data**).

### Statistical Analyses

Group comparisons for diversity metrics were conducted and graphed using R (version 3.6.3). α-Diversity (observed OTUs, Shannon index, and phylogenetic diversity) was compared by the decimal log-transformed relative abundance fecal microbiota between groups using the Wilcoxon rank-sum test (R package ‘microbiome version 1.9.19’). Group distances for β-diversity (weighted UniFrac metric and unweighted UniFrac metric) were generated with permutational analysis of variance (PERMANOVA) using 1000 Monte Carlo permutations (R package ‘phyloseq version 1.30.0’ and ‘vegan version 2.5.6’) and visualized with principal coordinate analysis (PCoA) plots. Receiver operating characteristic (ROC) curves of the random forest (RF) model were obtained using age, sex, and taxa for predicting CRC. The RF method was used with the RandomForestClassifier function of the sklearn package in Python (version 2.7.17), and a 10-fold cross-validation was applied to the training set. For ROC curves and the area under the curve (AUC), the pROC package in R was utilized. Discriminate taxa (>0.1% abundance) between the groups were identified using Welch’s t-test. Adjusted p-values controlling the false discovery rate (FDR) were reported where appropriate.

## Results

### Fecal Microbiota Composition in Colorectal Cancer (CRC) Patients and Control Subjects

**Figure 1:**
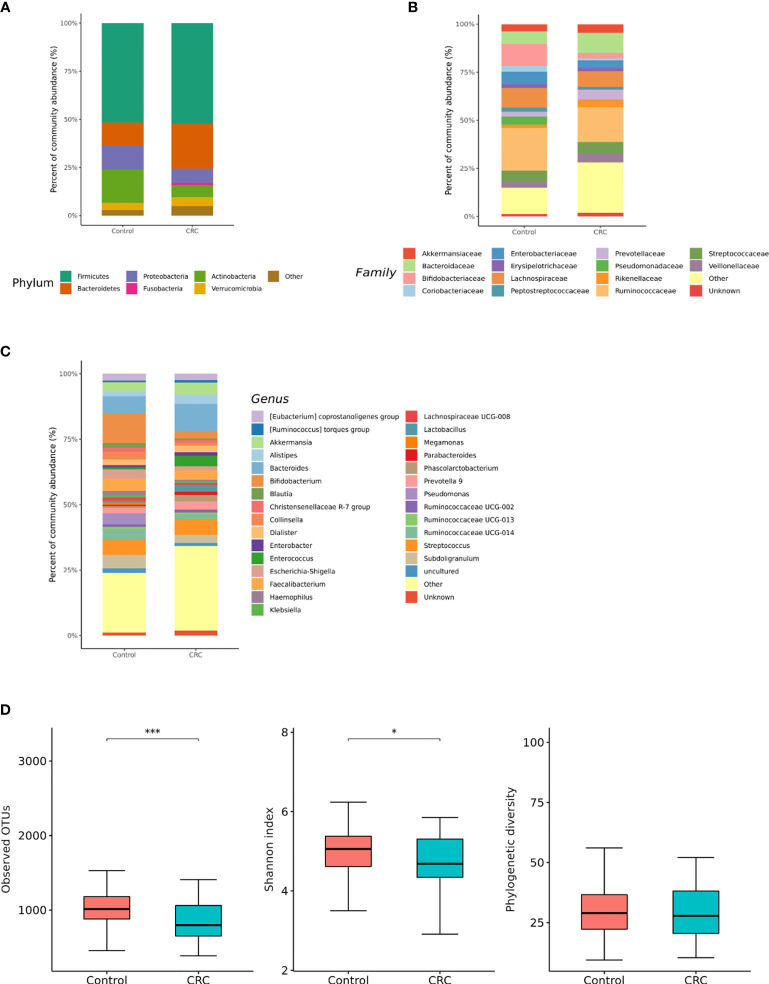
Relative abundance plots of the gut microbiota of control (n = 158) subjects and CRC subjects (n = 70) **(A)** at the phylum, **(B)** family, and **(C)** genus levels. **(D)** Boxplots of alpha diversity indices comparing CRC with control in the gut microbiota. ***p < 0.005 and *p < 0.05. CRC; colorectal cancer, OTU; operational taxonomic unit.

16S rRNA filtered gene sequences (**Supplementary Table 1**) were obtained from 228 stool samples (70 CRC subjects and 158 control subjects). The overall composition of the gut microbiota was altered in CRC subjects compared to that in the controls. Gut microbiota composition profiles were compared at the phylum, family, and genus levels (**Figures 1A–C**). CRC subjects showed a significant enrichment of Bacteroidetes phylum and depletion of Actinobacteria phylum (*p* < 0.001, **Table 1**). Within the phylum Bacteroidetes, relative enrichment was prominent for the family *Bacteroidaceae*, including genus *Bacteroides* (**Table 1**). Within Actinobacteria, relative depletion was prominent for the *Bifidobacterium* (*Bifidobacterium* genus) family in CRC subjects. Although there was no overall difference at the phylum level, several compositional changes were found at the family and genus levels. The relative abundance of the family *Clostridiaceae* 1 (genus *Clostridium sensu stricto* 1), *Family XIII (*genus *Family XIII* AD3011 group*)*, and *Erysipelotrichaceae* (genus *Erysipelotrichaceae* UCG-003) was lower in the CRC group than in the control group (**Table 1**). *Ruminococcus* I, *Ruminococcaceae* UCG-013 (family *Ruminococcaceae*), *Blautia [Eubacterium] hallii* group, and *Lachnospiraceae* NC2004 group (family *Lachnospiraceae*) were also significantly depleted in the CRC group (**Table 1**).

**Table 1:** Taxa showing a significant different in the abundance between CRC with control in the gut microbiota.

Taxon	t-statistic	Unadjusted *p-*value	Adjusted *p-*value	Reference
Phylum Firmicutes				
Family *Clostridiaceae 1*	-4.17	<0.001	**0.002**	(31)
Genus *Clostridium sensu stricto 1*	-4.17	<0.001	**0.004**	
Family *Family XIII*	-3.73	<0.001	**0.004**	(32)
Genus *Family XIII AD 3011 group*	-3.73	<0.001	**0.008**	
Family *Erysipelotrichaceae*	-3.50	<0.001	**0.016**	(31)
Genus *Erysipelotrichaceae UCG-003*	-3.50	0.001	**0.042**	
Genus *Turicibacter*	-2.89	0.004	0.129	(31)
Family *Ruminococcaceae*				(31, 33)
Genus *Ruminococcus 1*	-5.81	<0.001	**0.004**	(34, 35)
Genus *Ruminococcaceae UCG-013*	-3.78	<0.001	**0.004**	
Genus *Butyricicoccus*	-2.68	0.009	0.224	(36)
Genus *Subdoligranulum*	-2.59	0.012	0.286	
Family *Lachnospiraceae*				(31, 34)
Genus *Blautia*	-4.27	<0.001	**0.009**	(35)
Genus *[Eubacterium] hallii group*	-3.82	<0.001	**0.003**	(37)
Genus *Lachnospiraceae NC2004 group*	-3.45	<0.001	**0.003**	
Genus *[Eubacterium] ventriosum*	-3.08	0.004	0.117	(37)
Genus *[Ruminococcus] torques group*	-2.15	0.037	0.553	
Family *Peptostreptococcaceae*	-2.80	0.008	0.128	(31, 33)
Genus *Romboutsia*	-3.15	0.002	0.071	
Genus *Terrisporobacter*	-2.43	0.020	0.393	
Family *Christensenellaceae*	-2.76	0.007	0.124	(31)
Genus *Christensenellaceae R-7 group*	-2.76	0.007	0.207	
Family *Leuconostocaceae*	-2.77	0.008	0.124	(38)
Genus *Weissella*	-2.77	0.008	0.209	
Family *Lactobacillaceae*	-2.37	0.022	0.234	(31)
Genus *Lactobacillus*	-2.37	0.022	0.405	
Phylum Bacteriodetes	6.55	<0.001	**<0.001**	
Family *Bacteroidaceae*	5.26	<0.001	**0.002**	
Genus *Bacteroides*	5.26	<0.001	**0.004**	(34, 39)
Family *Marinifilaceae*	2.69	0.009	0.128	
Genus *Odoribacter*	2.69	0.009	0.227	(31, 33, 34, 39)
Family *Rikenellaceae*	2.47	0.015	0.185	(31)
Genus *Alistipes*	2.47	0.015	0.325	(33, 34)
Family *Tannerellaceae*	2.03	0.046	0.390	
Genus *Parabacteroides*	2.03	0.046	0.613	(34)
Phylum Actinobacteria	-5.82	<0.001	**<0.001**	
Family *Bifidobacteriaceae*	-5.93	<0.001	**0.002**	(31)
Genus *Bifidobacterium*	-5.93	<0.001	**0.004**	(34, 35)
Family *Eggerthellaceae*	-2.52	0.014	0.185	
Genus *Eggerthella*	-2.52	0.014	0.321	(33)
Family *Actinomycetaceae*	-2.36	0.021	0.234	
Genus *Actinomyces*	-2.36	0.021	0.405	(31)

The change of column (log2 fold) represents the multiplicative change in taxa abundance from CRC to control.

Negative numbers represent a trend of decreasing abundance in CRC group compared with control group. The data (p < 0.05) was provied as bold values.

**Figure 2:**
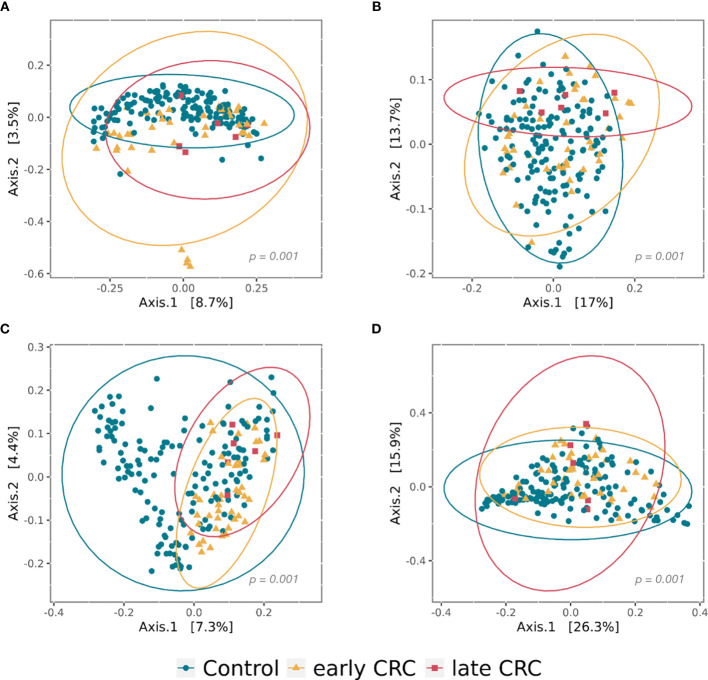
Principal coordinate analysis (PCoA) plots of beta diversity analysis of control, early CRC, and late CRC patients in the gut microbiota **(A–B)** and the gut microbe-derived extracellular vesicles **(C–D)**. Between-sample dissimilarities were measured by unweighted UniFrac distances **(A, C)** and weighted UniFrac distances **(B, D)**. Permutational multivariate analysis of variance (PERMANOVA) was performed to analyze statistical significance (p = 0.001).

**Figure 3:**
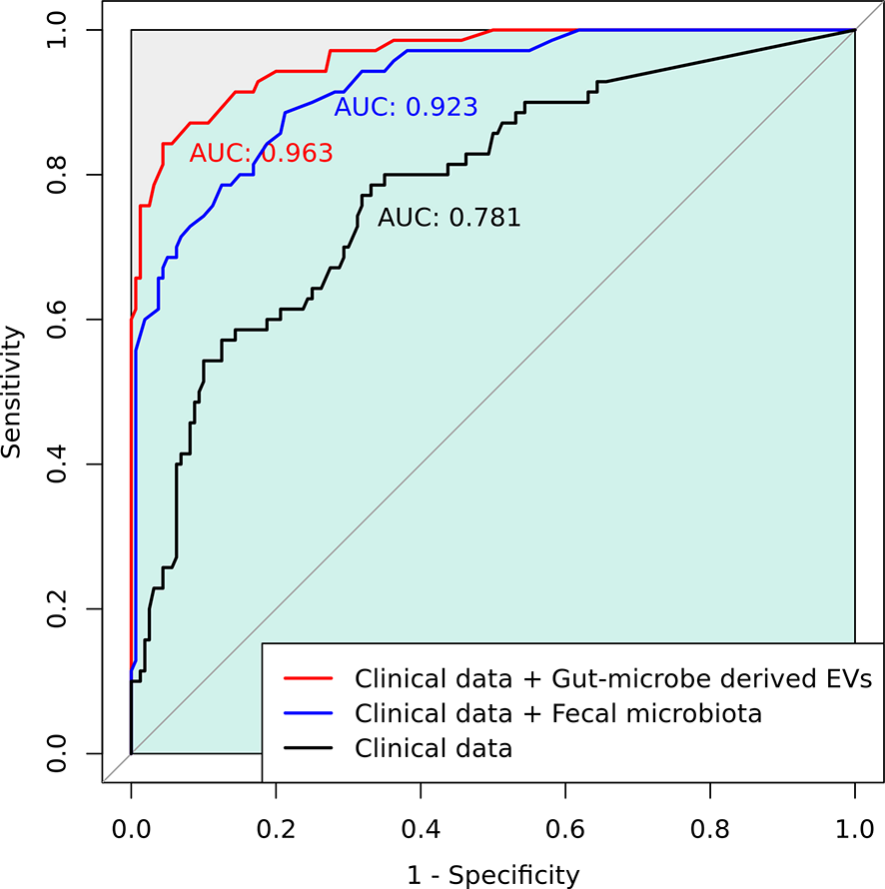
A combined receiver operating characteristic (ROC) analysis using clinical data and gut microbe-derived EVs.

Gut microbial community structure, assessed by richness and diversity, demonstrated a significantly lower richness and diversity in CRC subjects than in control subjects (**Figures 1D** and **2A, B**). A combined ROC analysis using clinical data and fecal microbiota revealed an AUC of 0.923 (**Figure 3**).

### Gut Microbe-Derived Extracellular Vesicles Composition in Colorectal Cancer (CRC) Patients and Control Subjects

**Figure 4:**
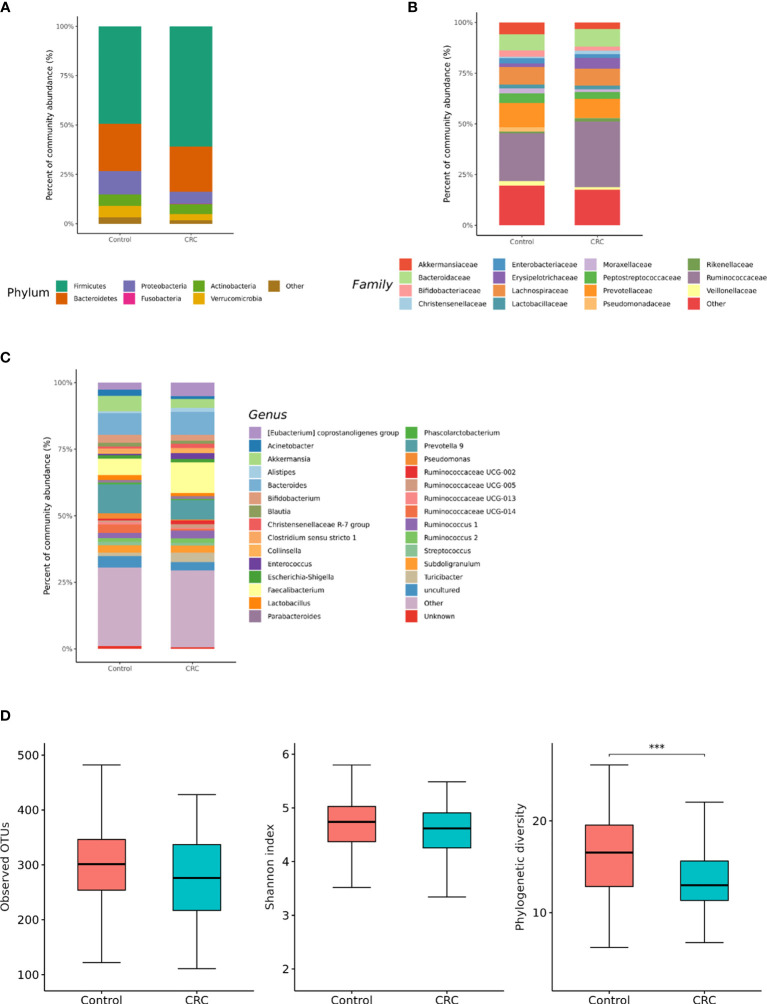
Relative abundance plots of gut microbe-derived extracellular vesicles of control (n = 158) subjects and CRC subjects (n = 70) **(A)** at the phylum, **(B)** family, and **(C)** genus levels. **(D)** Boxplots of alpha diversity indices comparing CRC with control in gut microbe-derived extracellular vesicles. ***p < 0.005. CRC; colorectal cancer, OTU; operational taxonomic unit.

We compared microbiota with gut microbe-derived EVs between CRC subjects and control subjects at the phylum, family, and genus levels (**Figures 4A–C****)**. CRC subjects showed a significant enrichment of Firmicutes (*p* = 0.008) and depletion of the Verrucomicrobia phylum (*p* = 0.002, **Table 2**). Within the Firmicutes phylum, the relative abundances of *Clostridiaceae* 1 (genus *Clostridium sensu stricto* 1), *Erysipelotrichaceae* (genus *Turicibacter*), *Peptostreptococcaceae* (genus *Romboutsia* and *Terrisporobacter*), *Veillonellaceae* (genus *Dialister*), *Staphylococcaceae* (genus *Staphylococcus*), and *Acidaminococcaceae* (genus *Phascolarctobacterium*) was lower in the control group (**Table 2**). Relative enrichment was prominent for the family *Erysipelotrichaceae* (genus *Catenibacterium, Erysipelotrichaceae* UCG-003), *Ruminococcaceae* (genus *Faecalibacterium, Ruminococcus 2*), *Lachnospiraceae* [genus *Blautia, (Eubacterium) hallii* group*, (Ruminococcus) torque* group*, Oribacterium, Dorea*] (**Table 2**). Within the Verrucomicrobia phylum, the *Akkermansiaceae* (genus *Akkermansia*) family was significantly lower in the CRC group than in the control group (**Table 2**). Within the Actinobacteria phylum, a significant enrichment of the family *Coriobacteriaceae* (genus *Collinsella*) was prominent (**Table 2**).

**Table 2:** Taxa showing a significant different in the abundance between CRC with control in the gut microbe-derived extracellular vesicles.

Taxon	t-statistic	Unadjusted *p*-value	Adjusted *p-*value
Phylum Firmicutes	3.16	0.002	**0.008**
Family *Clostridiaceae 1*	-3.36	0.001	**0.019**
Genus *Clostridium sensu stricto 1*	-3.36	0.001	**0.003**
Family *Erysipelotrichaceae*	3.28	0.001	**0.019**
Genus *Catenibacterium*	7.51	<0.001	**0.004**
Genus *Erysipelotrichaceae UCG-003*	4.41	<0.001	**0.004**
Genus *Turicibacter*	-2.00	0.043	0.541
Family *Ruminococcaceae*	4.22	<0.001	**0.002**
Genus *Faecalibacterium*	3.41	<0.001	**0.020**
Genus *Ruminococcus 1*	2.96	0.004	0.089
Genus *Ruminococcus 2*	2.23	0.028	**0.028**
Family *Lachnospiraceae*			
Genus *Blautia*	3.78	<0.001	**0.012**
Genus *[Eubacterium] hallii group*	5.04	<0.001	**0.004**
Genus *[Ruminococcus] torques group*	4.85	<0.001	**0.004**
Genus *Oribacterium*	3.79	<0.001	**0.012**
Genus *Dorea*	3.91	0.004	**0.018**
Genus *Lachnospiraceae UCG-008*	2.52	0.012	0.230
Family *Peptostreptococcaceae*	-3.25	0.003	**0.033**
Genus *Romboutsia*	-4.35	<0.001	**0.001**
Genus *Terrisporobacter*	-3.56	<0.001	**0.003**
Genus *Intestinibacter*	-3.07	0.004	0.092
Family *Veillonellaceae*	-6.10	<0.001	**0.002**
Genus *Dialister*	-6.10	<0.001	**0.004**
Family *Staphylococcaceae*	-4.61	<0.001	**0.002**
Genus *Staphylococcus*	-4.61	<0.001	**0.004**
Family *Acidaminococcaceae*	-3.28	0.001	**0.018**
Phylum Proteobacteria			
Family *Moraxellaceae*			
Genus *Enhydrobacter*	-2.15	0.034	0.476
Phylum Actinobacteria			
Family *Coriobacteriaceae*	4.78	<0.001	**0.004**
Genus *Collinsella*	4.78	<0.001	**0.007**
Phylum Verrucomicrobia	-3.77	<0.001	**0.002**
Family *Akkermansiaceae*	-3.77	<0.001	**0.004**
Genus *Akkermansia*	-3.77	<0.001	**0.012**

The change of column (log2 fold) represents the multiplicative change in taxa abundance from CRC to control.

Negative numbers represent a trend of decreasing abundance in CRC group compared with control group. The data (p < 0.05) was provied as bold values.

The gut microbe-derived extracellular vesicle composition and gut microbial community structure, assessed by richness and diversity, demonstrated a significant difference in richness and diversity between the CRC and control subjects (**Figures 2C, D**, **4D**). A combined ROC analysis using clinical data and gut microbe-derived EVs revealed an AUC of 0.963 (**Figure 3**).

### Differences in the Microbial Composition According to the Colorectal Cancer (CRC) Stage

**Figure 5:**
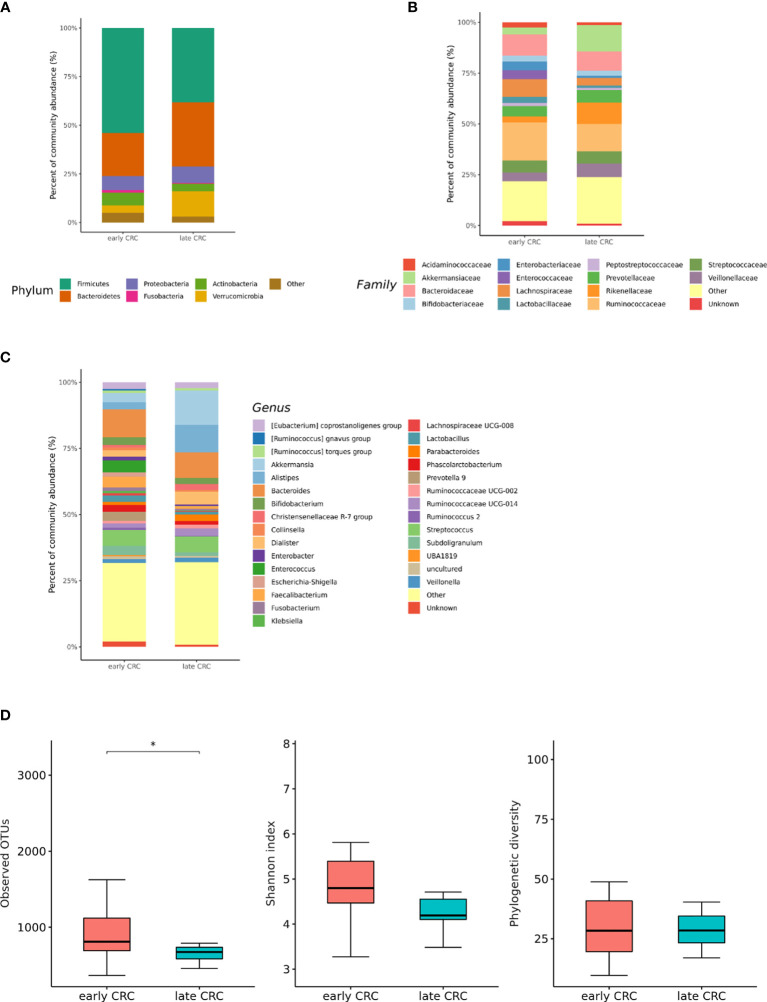
Relative abundance plots of the gut microbiota of early CRC (n = 62) and late CRC (n=8) **(A)** at the phylum, **(B)** family, and **(C)** genus levels. **(D)** Boxplots of alpha diversity indices comparing late CRC with early CRC in the gut microbiota. *p < 0.05. CRC; colorectal cancer, OTU; operational taxonomic unit.

We analyzed the differences in the microbial composition according to the CRC stage. Out of the 70 patients with CRC, 62 patients were in stages I, II, or III, defined as early CRC, and 8 patients were in stage IV, defined as late CRC. In fecal microbiota, late CRC subjects tended to have an enrichment of Bacteroidetes and depletion of Actinobacteria compared with early CRC subjects (**Figures 5A–C**). In fecal microbiota, within Bacteroidetes, relative enrichment was prominent for the family *Marinifilaceae* (genus *Odoribacter*) and *Rikenellaceae* (genus *Alistipes*) in late CRC subjects than in early CRC subjects before adjustment (**Supplementary Table 2****)**. *Odoribacter* and *Alistipes* genera were significantly increased in CRC subjects compared to control subjects (**Table 1**). Microbial composition changes in control and CRC subjects and those of early and late CRC subjects were similar. Relative depletion was prominent for the family *Prevotellaceae* (genus *Prevotella 9*) in late CRC subjects. Within Firmicutes, relative depletion was prominent for the family *Ruminococcaceae* (genus *Butyricicoccus*) in late CRC subjects (**Supplementary Table 2****)**. *Butyricicoccus* was significantly decreased in CRC subjects compared to control subjects (**Table 1**).

**Figure 6:**
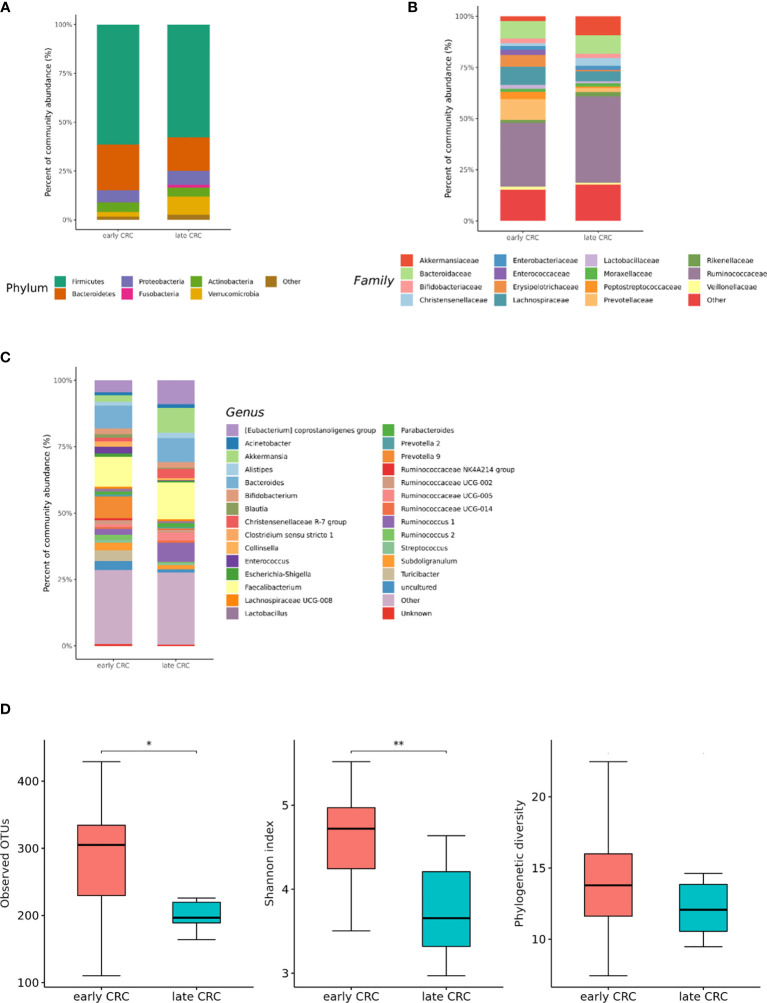
Relative abundance plots of gut microbe-derived extracellular vesicles of early CRC (n = 62) and late CRC (n=8) **(A)** at the phylum, **(B)** family, and **(C)** genus levels. **(D)** Boxplots of alpha diversity indices comparing late CRC with early CRC in gut microbe-derived extracellular vesicles. **p < 0.01 and *p < 0.05. CRC; colorectal cancer, OTU; operational taxonomic unit.

In the microbiota with gut microbe-derived EVs, relative enrichment was prominent for the family *Rikenellaceae* (genus *Alistipes*) in late CRC subjects compared to early CRC subjects before adjustment (**Figures 6A–C** and **Supplementary Table 3****)**. Within Firmicutes, relative enrichment was prominent for the family *Acidaminococcaceae* (genus *Phascolarctobacterium*), and relative depletion was prominent for the family *Lactobacillaceae* (genus *Lactobacillus*) in late CRC subjects compared to early CRC subjects before adjustment (**Supplementary Table 3****)**.

CRC fecal microbiota and CRC gut microbe-derived EV composition and gut microbial community structure, assessed by richness and diversity, demonstrated a significantly different richness and diversity in late CRC subjects than in early CRC subjects. (**Figures 2A, B**, **5D** and **6D**).

### Differences in the Microbial Composition According to the Colorectal Cancer (CRC) Location

**Figure 7:**
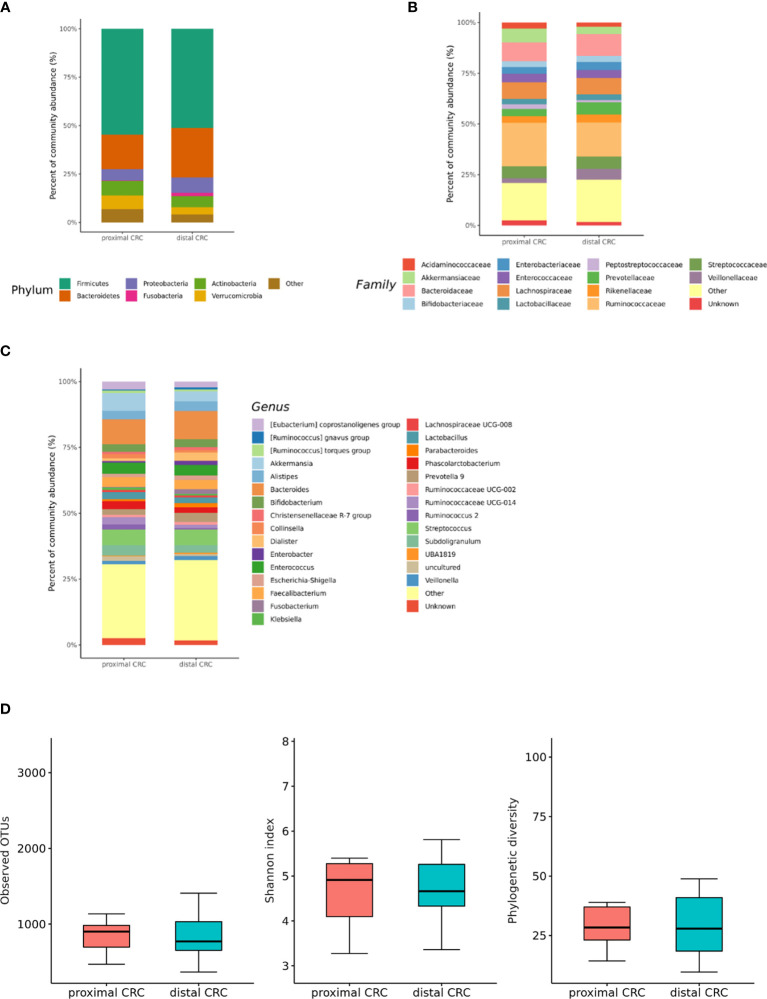
Relative abundance plots of the gut microbiota of proximal CRC (n = 20) versus distal CRC (n = 50) **(A)** at the phylum, **(B)** family, and **(C)** genus levels. **(D)** Boxplots of alpha diversity indices comparing distal CRC with proximal CRC in the gut microbiota. CRC; colorectal cancer, OTU; operational taxonomic unit.

**Figure 8:**
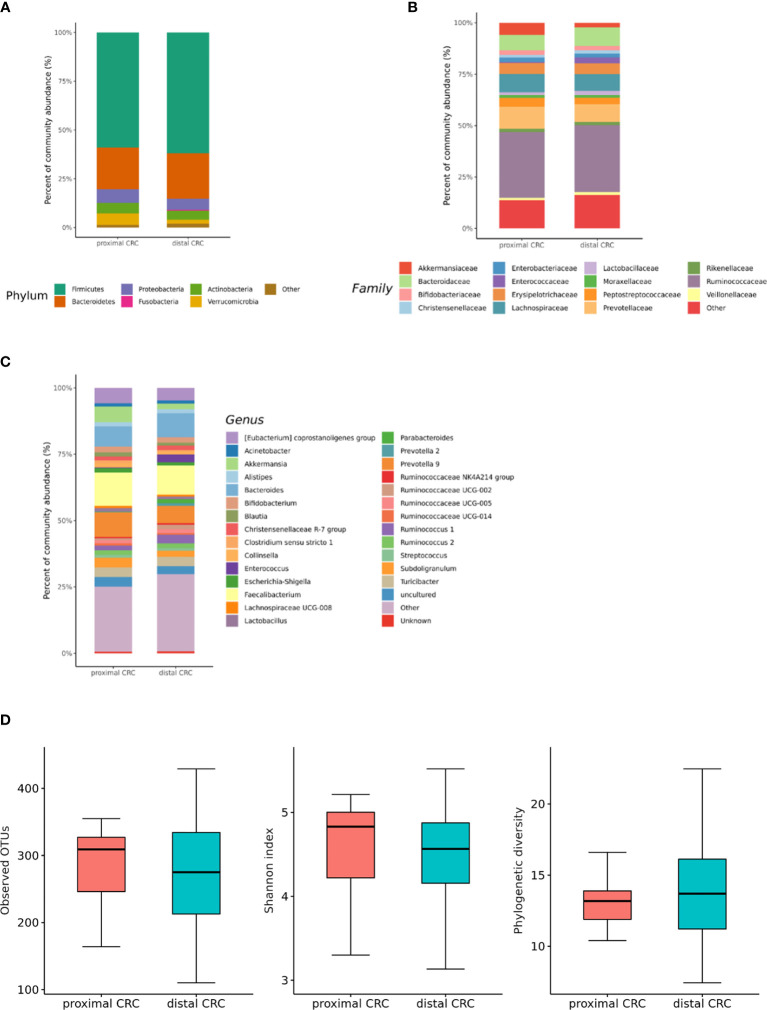
Relative abundance plots of gut microbe-derived extracellular vesicles of proximal CRC (n = 20) versus distal CRC (n = 50) **(A)** at the phylum, **(B)** family, and **(C)** genus levels. **(D)** Boxplots of alpha diversity indices comparing distal CRC with proximal CRC in gut microbe-derived extracellular vesicles. CRC; colorectal cancer, OTU; operational taxonomic unit.

We also analyzed the differences in the microbial composition according to the CRC location. Of the 70 patients with CRC, 20 had proximal CRC and 50 had distal CRC. There were no significant differences in gut microbial composition changes between the proximal and distal CRC subjects (**Figures 7A–C****)**. In microbiota with gut microbe-derived EVs, within the Firmicutes phylum, relative depletion was prominent for family *Ruminococcaceae* (genus *Ruminococcus* 2) in the distal CRC subjects compared to proximal CRC subjects (**Figures 8A–C** and **Supplementary Table 4**). Alpha diversity was not different between distal CRC and proximal CRC in both fecal microbiota and gut microbe-derived EVs (**Figures 7D** and **8D**). Microbial community structure was assessed by evenness with both stool and microbe-derived EVs, but distal CRC subjects did not differ from proximal CRC subjects.

## Discussion

The human gut microbiota comprises trillions of microbes, and 20% of human malignancies are caused by dysbiosis (40). In the past decade, metagenomic sequencing has broadened our understanding of microbial composition. The gut microbiota plays an important role in CRC initiation by chronic inflammation, which affects the intestinal epithelial cells. Fecal and mucosal microbial changes in CRC patients have been studied; however, no consistent patterns among these studies have been observed (6, 41–43).

This study analyzed stool profiling to identify the changes in microbial composition in CRC patients in South Korea. CRC subjects showed a significant enrichment of Bacteroidetes phylum and depletion of Actinobacteria phylum. At the genus level, the relative abundances of *Bacteroides*, *Odoribacter*, *Alistipes*, and *Parabacteroides* were higher in the CRC patients than in the healthy controls. The top five genera dominant in CRC patients were *Bifidobacterium*, *Ruminococcus* 1, *Blautia*, *Clostridium sensu stricto* 1, and *the Eubacterium hallii* group. In previous studies, patients with CRC had higher proportions of pathogenic bacteria, including *Bacteroides*, *Odoribacter*, and *Alistipes*, and fewer *Bifidobacterium*, *Ruminococcus*, and *Blautia*, similar to our results (34, 44). Putrefactive bacteria such as *Alistipes* and *Bacteroides* can produce short-chain fatty acids and promote chronic intestinal inflammation (45). The decrease in *Bifidobacterium*, *Ruminococcus*, and *Blautia*, which act as potential probiotics and function as antibacterial agents, could enhance CRC development (35). Additionally, we found that the richness and diversity of the fecal microbiota of patients with CRC were significantly lower than those of the healthy controls. Dongmei et al. also suggested that alpha diversity was significantly higher in the control subjects than in the CRC subjects (46). The difference in fecal microbial composition between the CRC and control subjects suggests that fecal microbiota could be a possible diagnostic biomarker in the future.

To discover a new effective biomarker of CRC stages, we also analyzed the different genera between early and late CRC subjects. We found that *the Odoribacter* and *Alistipes* genera were significantly increased not only in late CRC subjects compared to early CRC subjects but also in CRC subjects compared to control subjects. *Odoribacter* and *Alistipes* genera could be novel biomarkers for diagnosing CRC and predicting CRC stages. Genus *Alistipes* is an emerging gut bacteria related to inflammation, cancer, and mental health, as per a recent review (45). *Alistipes* evokes colitis and proximal colon cancers in IL10-/- mice (47). In addition, *Butyricicoccus* was significantly decreased in CRC subjects compared to control subjects. *Butyricicoccus* is a gut butyrate-producing bacterium that improves the clinical outcome of CRC by administration of *Butyricicoccus pullicaecorum*, as demonstrated in a previous mouse model (48). This result suggests that *Butyricicoccus* might be a novel pharmacological agent to prevent CRC pathogenesis.

To the best of our knowledge, there are few studies on the composition of gut microbe-derived EVs in CRC patients compared with controls (23). In principal coordinate analysis, the microbial community structure of the CRC gut microbe-derived EVs was different from that of the control subjects. This study showed that the composition of gut microbe-derived EVs differed significantly from that of fecal microbiota. These results indicate that gut microbe-derived EVs might be a better novel biomarker than fecal microbiota. Gut microbe-derived EVs could play an important role in transferring proteins and nucleic acids from cells to other cells as nanocarriers (49). Tumors and other cells secrete EVs, and tumor-derived EVs can stimulate tumor progression, invasion, angiogenesis, and metastasis (50). Gut microbe-derived EVs in CRC patients may play a pivotal role in tumorigenesis. Several studies have examined EVs in CRC patients and animal models of IBD (51, 52). In the present study, *Clostridium sensu stricto* 1, *Turicibacter*, *Romboutsia*, *Terrisporobacter*, *Dialister*, *Staphylococcus*, *Phascolarctobacterium*, and *Akkermansia-*derived EVs could be effective therapeutic candidates for CRC treatment. Additionally, *Catenibacterium*, *Erysipelotrichaceae* UCG-003, *Faecalibacterium*, *Ruminococcus* 2, *Blautia [Eubacterium] hallii* group, *Ruminococcus torques* group, *Oribacterium*, *Dorea*, and *Collinsella-derived* EVs could be novel biomarkers for CRC diagnosis. Although it is difficult to determine whether the differentially relative abundance of microbe-derived EVs between CRC patients and healthy controls, the development of targeted analysis of cancer-derived EVs with specimens for the diagnosis and treatment monitoring in clinical settings has gained attention and challenges in recent years (53). We performed ROC analysis and established a prediction model for CRC diagnosis using gut-derived EVs compared to fecal microbiota. The microbiota enhanced the performance for predicting CRC diagnosis compared to the model with only clinical data. Among the microbiota data, gut-derived EVs outperformed fecal microbiota.

According to the CRC stage, *Alistipes*-derived EVs were significantly increased not only in late CRC subjects compared to early CRC subjects but also in CRC subjects compared to control subjects. *Alistipe*-derived EVs could be novel biomarkers for diagnosing CRC and predicting CRC stages. *Phascolarctobacterium-*derived EVs were significantly increased, and *Lactobacillacea-*derived EVs were significantly decreased in late CRC subjects compared to early CRC subjects. Gut microbe-derived EVs in CRC patients could play an important role in the development and growth of CRC.

Studies have identified metagenomic biomarkers for CRC formation (54). We analyzed the differences in the microbial composition according to the CRC location. In the microbiota with gut microbe-derived EVs, *Ruminococcus* 2 was lower in the distal CRC subjects than in the proximal CRC subjects. The microenvironment could affect the pathogenesis of CRC development, and enrichment of *Ruminococcus* 2 could be associated specifically with proximal CRC development. However, the difference according to the CRC stage was more significant than that according to the CRC location.

Our study has several limitations. First, although our research team has approved the EV isolation method in previous studies, ultracentrifugation with a relatively low number of turns could influence our results. Second, future studies for dynamic light scattering (DLS) or transmission electron microscopy (TEM) images to trace the size of vesicles are warranted. Third, we discovered candidate microbe-derived EVs for CRC prediction, but we did not validate them. Further study for validation with PCR on the target taxa will be warranted to quantify the microbe-derived EVs for CRC prediction.

In summary, this is a report on the metagenomic analysis of gut microbe-derived EVs in CRC patients. Profiling of microbe-derived EVs may offer a novel biomarker for detecting and predicting the prognosis of CRC.

## Data Availability Statement

The datasets presented in this study can be found in online repositories. The names of the repository/repositories and accession number(s) can be found below: https://www.ncbi.nlm.nih.gov/, SRR15182562–SRR15182631, https://www.ncbi.nlm.nih.gov/, SRR15182632–SRR15182701, https://www.ncbi.nlm.nih.gov/, SRR15056567–SRR15056766, https://www.ncbi.nlm.nih.gov/, SRR15056787–SRR15056992, https://www.ncbi.nlm.nih.gov/, SRR15244175-SRR15244358, https://www.ncbi.nlm.nih.gov/, SRR15245161-SRR15245345, https://www.ncbi.nlm.nih.gov/, SRR15204197-SRR15221118, https://www.ncbi.nlm.nih.gov/, SRR15243500-SRR15243683.

## Ethics Statement

The studies involving human participants were reviewed and approved by Institutional Review Board of Seoul National University Bundang Hospital (IRB No: B-1708/412–301). The patients/participants provided their written informed consent to participate in this study.

## Author Contributions

Guarantor of the article: YP. Development of study concept and design: JP, HY, CS, and YP. Study supervision: HY, CS, YP, NK, and DL. Acquisition, analysis, and interpretation of data: N-EK, JP, HY, CS, JYP, CC, JK, YP, NK, DL, Y-KK, T-SS, and JY. Statistical analysis: N-EK. Drafting of the manuscript: JP. Critical revision of the manuscript for important intellectual content: HY, CS, and YP. All authors contributed to the article and approved the submitted version.

## Funding

This research was supported by the Bio & Medical Technology Development Program of the National Research Foundation (NRF), funded by the Ministry of Science and ICT (2017M3A9F3047495).

## Conflict of Interest

Authors Y-KK, T-SS and JY were employed by Institute of MD Healthcare Inc.

The remaining authors declare that the research was conducted in the absence of any commercial or financial relationships that could be construed as a potential conflict of interest.

## Publisher’s Note

All claims expressed in this article are solely those of the authors and do not necessarily represent those of their affiliated organizations, or those of the publisher, the editors and the reviewers. Any product that may be evaluated in this article, or claim that may be made by its manufacturer, is not guaranteed or endorsed by the publisher.

## Abbreviations

CRC, colorectal cancer; EV, extracellular vesicle; PD-1, programmed death receptor-1; CTLA-4, cytotoxic T-lymphocyte associated protein 4; out, operational taxonomic unit; PCoA, principal coordinate analysis; ROC, receiver operating characteristic; RF, random forest; AUC, area under the curve; IBD, inflammatory bowel disease.
