# Supplementary material for: Fecal Microbiota and Gut Microbe-Derived Extracellular Vesicles in Colorectal Cancer
Source: Front Oncol. 2021 Sep 14;11:650026. doi: 10.3389/fonc.2021.650026 (PMC8477046; doi:10.3389/fonc.2021.650026)
Supplement: Supplementary file 1 [file Table_1.docx]

| **Supplementary Table 1.** Baseline characteristics | | |  |
| --- | --- | --- | --- |
| **Variables** | **Control group (n=158)** | **Colorectal cancer group (n=70)** | ****p* value** |
| Age (years) | 52.5 ± 8.5 | 63.1 ± 9.1 | <0.001 |
| Male | 28 (17.7%) | 46 (65.7%) | <0.001 |
| Smoking history | 5 (3.2%) | 34 (50.0%) | <0.001 |
| Alcohol history | 52 (32.9%) | 33 (49.3%) | 0.031 |
| Body mass index (kg/m^2^) | 25.9 ± 3.5 | 24.0 ± 3.8 | 0.001 |
| Variables are expressed as mean ± SD or n (%). | |  |  |
| **p* value for comparing control group with colorectal cancer group. | | |  |

| **Supplementary table 2**. Taxa showing a significant different abundance between late CRC with early CRC in the gut microbiota. | | | |
| --- | --- | --- | --- |
| Taxon | t-statistic | Unadjusted *p* value | Adjusted *p* value |
| Phylum Bacteriodetes |  |  |  |
| Family *Marinifilaceae* | 2.42 | 0.039 | 0.562 |
| Genus *Odoribacter* | 2.42 | 0.039 | 0.779 |
| Family *Rikenellaceae* | 2.81 | 0.037 | 0.550 |
| Genus *Alistipes* | 2.81 | 0.037 | 0.779 |
| Family *Prevotellaceae* | -2.68 | 0.039 | 0.562 |
| Genus *Prevotella 9* | -2.68 | 0.039 | 0.562 |
| Phylum Firmicutes |  |  |  |
| Family *Ruminococcaceae* |  |  |  |
| Genus *Butyricicoccus* | -2.33 | 0.039 | 0.779 |
| The change of column (log2 fold) represents the multiplicative change in taxa abundance from late CRC to early CRC group. | | | |
| Negative numbers represent a trend of decreasing abundance in late CRC group compared with early CRC group. | | | |

| **Supplementary table 3.** Taxa showing a significant different abundance between late CRC with early CRC in the gut microbe-derived extracellular vesicles. | | | |
| --- | --- | --- | --- |
| Taxon | t-statistic | Unadjusted *p* value | Adjusted *p* value |
| Phylum Bacteriodetes |  |  |  |
| Family *Rikenellaceae* | 3.15 | 0.024 | 0.322 |
| Genus *Alistipes* | 3.15 | 0.024 | 0.529 |
| Phylum Firmicutes |  |  |  |
| Family *Erysipelotrichaceae* | -2.98 | 0.010 | 0.165 |
| Family *Lactobacillaceae* | -3.63 | 0.011 | 0.176 |
| Genus *Lactobacillus* | -3.63 | 0.011 | 0.303 |
| Family *Acidaminococcaceae* | 2.76 | 0.048 | 0.550 |
| Genus *Phascolarctobacterium* | 2.76 | 0.048 | 0.762 |
| The change of column (log2 fold) represents the multiplicative change in taxa abundance from late CRC to early CRC group. | | | |
| Negative numbers represent a trend of decreasing abundance in late CRC group compared with early CRC group. | | | |

| **Supplementary table 4.** Taxa showing a significant different abundance between distal CRC with proximal CRC in the gut microbe-derived extracellular vesicles. | | | |
| --- | --- | --- | --- |
| Taxon | t-statistic | Unadjusted *p* value | Adjusted *p* value |
| Phylum Firmicutes |  |  |  |
| Family *Ruminococcaceae* |  |  |  |
| Genus *Ruminococcus 2* | -2.68 | 0.016 | 0.433 |
| The change of column (log2 fold) represents the multiplicative change in taxa abundance from distal CRC to proximal CRC group. | | | |
| Negative numbers represent a trend of decreasing abundance in distal CRC group compared with proximal CRC group. | | | |
